# Automated road surface classification in OpenStreetMap using MaskCNN and aerial imagery

**DOI:** 10.3389/fdata.2025.1657320

**Published:** 2025-08-13

**Authors:** R. Parvathi, V. Pattabiraman, Nancy Saxena, Aakarsh Mishra, Utkarsh Mishra, Ansh Pandey

**Affiliations:** School of Computer Science and Engineering, Vellore Institute of Technology – Chennai Campus, Chennai, Tamil Nadu, India

**Keywords:** road surface classification, OpenStreetMap (OSM), machine learning, aerial imagery, MaskCNN, segmentation masks, model calibration, PyTorch Lightning

## Abstract

**Introduction:**

OpenStreetMap (OSM) road surface data is critical for navigation, infrastructure monitoring, and urban planning but is often incomplete or inconsistent. This study addresses the need for automated validation and classification of road surfaces by leveraging high-resolution aerial imagery and deep learning techniques.

**Methods:**

We propose a MaskCNN-based deep learning model enhanced with attention mechanisms and a hierarchical loss function to classify road surfaces into four types: asphalt, concrete, gravel, and dirt. The model uses NAIP (National Agriculture Imagery Program) aerial imagery aligned with OSM labels. Preprocessing includes georeferencing, data augmentation, label cleaning, and class balancing. The architecture comprises a ResNet-50 encoder with squeeze-and-excitation blocks and a U-Net-style decoder with spatial attention. Evaluation metrics include accuracy, mIoU, precision, recall, and F1-score.

**Results:**

The proposed model achieved an overall accuracy of 92.3% and a mean Intersection over Union (mIoU) of 83.7%, outperforming baseline models such as SVM (81.2% accuracy), Random Forest (83.7%), and standard U-Net (89.6%). Class-wise performance showed high precision and recall even for challenging surface types like gravel and dirt. Comparative evaluations against state-of-the-art models (COANet, SA-UNet, MMFFNet) also confirmed superior performance.

**Discussion:**

The results demonstrate that combining NAIP imagery with attention-guided CNN architectures and hierarchical loss functions significantly improves road surface classification. The model is robust across varied terrains and visual conditions and shows potential for real-world applications such as OSM data enhancement, infrastructure analysis, and autonomous navigation. Limitations include label noise in OSM and class imbalance, which can be addressed through future work involving semi-supervised learning and multimodal data integration.

## Introduction

Precise road quality classification is an important prerequisite for the work of (intelligent) navigation systems, autonomous driving, and urban infrastructure planning. OSM (OpenStreetMap), a popular open-source mapping service, has surface tags that are user-contributed labels that are assigned to roadways. However, studies have demonstrated that OSM data usually suffer from incompleteness, inconsistency, and antiqueness (Barrington-Leigh and Millard-Ball, 2017); therefore, automated validation of OSM data is necessary. Road-type Road extraction classification of road types is a related field of study in aerial imagery. Conventional approaches include spectral analysis, handcrafted feature extraction and rule-based classification. However, these methods find it difficult to handle lighting variations, occlusions, and heterogeneous terrains. Recent progress in deep learning techniques such as convolutional neural networks (CNNs) has shown promising results in remote sensing applications (Maggiori et al., 2017; Sherrah, 2016).

### Contributions of this study

In this study, we introduce a deep learning approach to classify road surfaces using NAIP aerial imagery and OSM surface labels. Our contributions include the following:

- Data Integration and Preprocessing- Matching OSM road surface descriptions to NAIP imagery to create high quality training data.
- Analysis of Road Color Based on Spectral Characteristics and Texture—using spectral, color, and texture information to improve classification accuracy.
- Hierarchical Loss Model—A CNN-based segmentation model utilizing hierarchical loss functions to distinguish visually similar road surfaces is adopted.
- Model Calibration and Testing—Tuning and calibration of the model to enhance model performance and robustness across locations with different climates.

### Structure of the paper

The remainder of this paper is structured as follows:

- **Related work section** reviews previous research on road surface classification and geospatial deep learning.
- **Dataset description and preprocessing section** details the dataset, preprocessing pipeline, and feature extraction techniques.
- **Proposed methodology** describes the segmentation model architecture and hierarchical loss optimization.
- **Experimental result section** presents experimental results and comparisons with existing methods.
- **Conclusion section** concludes the study and discusses future research directions.

## Related work

The problem of road surface type classification has been considered in numerous ways, such as manual annotation, rule-based classification, machine learning, and deep learning methods. In this section, we review previous studies related to road surface detection using remote sensing, OpenStreetMap confirmation, and geospatial-deep learning methods.

### Road surface classification using remote sensing

High-resolution aerial imagery and satellite data have been extensively applied for road-surface classification. Traditional approaches include spectral analysis, feature extraction based on texture, and handcrafted classifiers. For example, spectral indices and texture measures have been employed in the initial methods to distinguish different road types with mixed success owing to mixed-pixel effects. Road segmentation has achieved compelling performance gains with the advent of deep learning, particularly via convolutional neural networks (CNNs). A typical example is the study by Zhang and Peng (2018), who presented a GL-Dense-U-Net model to extract roads from high-resolution remote sensing images, and improved performance results over classical methods. Similarly, Abdollahi et al. (2020) proposed an improved deep convolutional encoder–decoder (derived from SegNet) in combination with the ELU activation function to automatically segment road classes from high-resolution remote sensing images, to improve accuracy.

### OpenStreetMap (OSM) validation and road surface mapping

OSM is a useful crowdsourced mapping service, but its road-surface information is usually incomplete and incoherent. Haklay (2010) analyzed the accuracy of OSM road networks and discovered that surface tagging is often outdated or lacking. Fonte et al. (2020) examined the verification and improvement of OSM road-surface data using high-resolution satellite imagery and machine learning. To make the OSM compatible with remote sensing, Maggiori et al. (2019) proposed an automatic OSM validation method that compared road surfaces extracted from satellite images with OSM annotations. Their research showed that machine learning classifiers with aerial imagery as input were able to amend erroneous or absent road labels. Barrington-Leigh and Millard-Ball (2017) also studied the quality and consistency of OSM data and found regional differences in road surface annotations, as well as the availability and completeness of data at the global level.

### Deep learning for road segmentation and classification

The application of deep-learning models has resulted in the classification of road surfaces into new heights. Shelhamer et al. (2015) were the first to introduce fully convolutional networks (FCNs) for semantic segmentation, laying the groundwork for road extraction models. Based on this, Ronneberger et al. (2015) proposed U-Net as a segmentation baseline for roads using remote sensing. To improve classification accuracy, multi-spectral researchers have used by methods. Mnih and Hinton (2010) introduced an RGB+ near-infrared (NIR) CNN model that provided better performance in road detection in shadowed or occluded areas than the RGB model. Similarly,(Maggiori et al. 2017) presented a deep multi-scale method to model local and global road surface properties. Hierarchical loss has also been recently studied for robust training. Xu et al. (2018) proposed a hierarchical loss function that can improve the discrimination of visually similar road types. Additionally, Zhang (2017) explored attention-based CNN models trained on features relevant to the road to improve the model performance.

### Limitations of existing approaches

Despite the progress made, existing pavement-type classification models encounter some important challenges.

- Data quality problems: Labels in OSM, for instance, are frequently incomplete and require manual correction or automatic validation (Fonte et al., 2020).
- Spectral confusion: The same Road Type (for example, gravel vs. concrete) can look the same in aerial images, making classification difficult (Zhang and Peng, 2018).
- Cross-region generalization: Most deep-learning-based models fail to generalize training from one region to another with different lighting conditions and other factors (Bengio, 2012).

Our study addressed these challenges by combining NAIP imagery with OSM labels, leveraging hierarchical loss functions, and fine-tuning CNN architectures for robust road classification.

In recent years, superior deep learning architectures have emerged for road extraction and classification. Mei et al. (2021) were the first to introduce a Connectivity Attention Network (COANet) with a coarse-to-fine pipeline with context-enhanced connective modules to preserve road connectivity and diminution adipose scale. The model was more generalized, and able to deal with continuity, while using satellite images. Alternatively, Wang et al. (2024) proposed a Multiscale and Multidirection Feature Fusion Network (MMFFNet), which aimed to capture often overlooked directional and hierarchical features that increased detection accuracy in areas with increased heterogeneous complexity of roads (Wang et al., 2024). The SA-UNet model (Wu et al., 2024) operated on a classic U-Net backbone, introducing spatial attention nodes to highlight the salient features necessary for successful extraction of travel areas deemed as roads. Collectively, all of the studies in this section improved road extraction, but in their own respective ways.

## Dataset description and preprocessing

In this section, the processes of dataset selection, pre-processing pipeline, and feature extraction methods related to road surface classification are outlined. We employed NAIP high-resolution aerial imagery and OSM road surface labels in our method and used advanced data pre-processing techniques to improve the robustness and accuracy of the output.

### Dataset and data sources

We leveraged aerial imagery data from the National Agriculture Imagery Program (NAIP) and road surface labels from OpenStreetMap (OSM) to create an accurate and scalable road surface classification model.

#### NAIP aerial imagery

**Figure 1:**
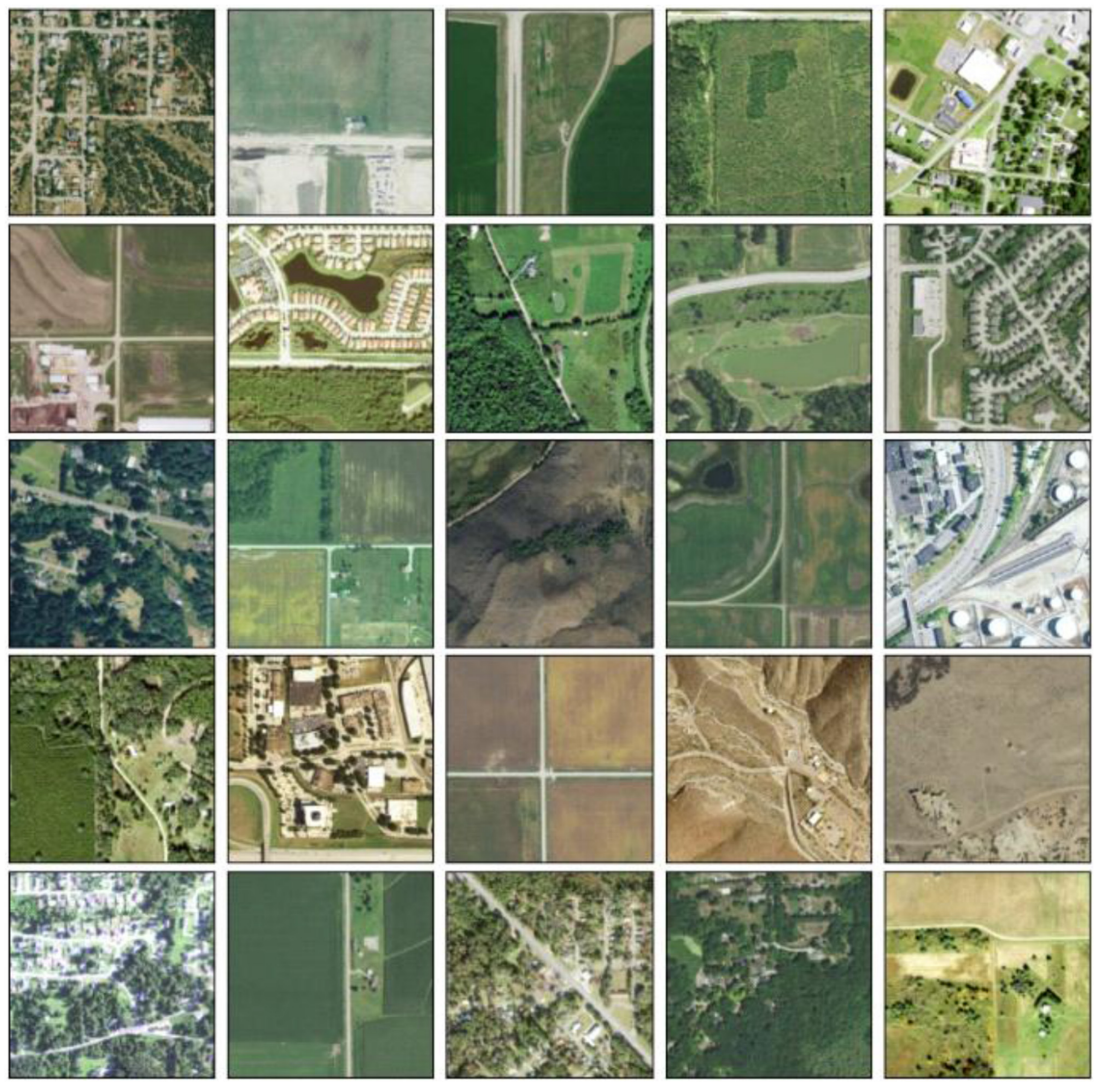
Example of NAIP imagery showing variations in road surfaces.

The NAIP data contain multi-band (red, green, blue, near infrared–NIR) high-resolution (1-meter per pixel) multispectral images. By capturing the surface reflectance properties, these spectral bands allow discrimination between paved and non-paved roads. The dataset encompasses urban, suburban, and rural domains to ensure inclusiveness, as shown in Figure 1.

#### OSM road surface labels

OpenStreetMap (OSM) is a crowdsourced mapping tool with surface labels, including asphalt, concrete, gravel, and dirt. However, OSM tags may contain missing or inconsistent data, which requires data validation (Singh et al., 2023).

### Data preprocessing

To align NAIP imagery with OSM road labels, we perform the following steps:

- Georeferencing and cropping: Georeference NAIP imagery to OSM and crop patches that contain roads.
- Surface labeling: Extracting OSM road segments with assigning surface labels.
- Transformation and noise filtering: The low-confidence OSM tags are removed, inconsistencies are corrected, and missing tags are infilled by spatial interpolation.
- Data augmentation: Use of rotation, flipping, adjusting brightness, and adding Gaussian noise to help overcome bulky malls and highways make the model general and robust.
- Patch normalization: The pixel intensity between images is normalized to ensure that they have consistent feature representations.
- Class balancing: Handles the problem of imbalanced distribution of labels through oversampling and generates synthetic data for minority classes.

**Figure 2:**
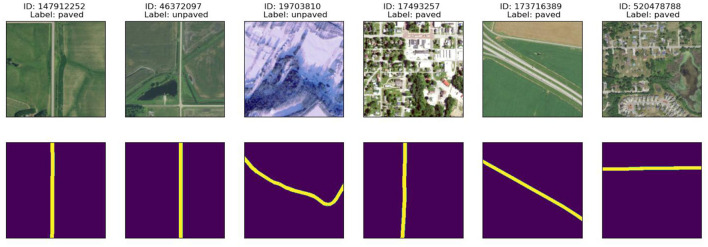
Examples of NAIP aerial imagery **(top row)** and their corresponding road surface masks **(bottom row)**, labeled as either paved or unpaved. These patches are representative of the dataset used during training and demonstrate the diversity and clarity in segmentation targets.

An excerpt of the patches from the NAIP imagery and their segmentation is shown in Figure 2. Each column represents the sample image, surface label (paved/unpaved), and predicted mask of the model. These visualizations demonstrate the variability in road appearance and demonstrate that the pre-processing pipeline, specifically georeferencing, mask alignment, and augmentation, retained both the visual and spatial quality of the road surfaces irrespective of terrain.

Table 1 shows the number of road segments per surface class, as originally downloaded from OSM, the number removed due to low confidence or ambiguous labels, and the number interpolated during preprocessing. This indicates the degree of data curation performed prior to training, as well as identifying the distribution bias in crowdsourced data.

**Table 1:** Road segment counts before and after cleaning.

**Surface class**	**Original OSM segments**	**Discarded (low confidence)**	**After cleaning**	**Patches auto-filled (interpolated)**
Asphalt	14,238	1,225	13,013	812
Concrete	3,517	402	3,115	289
Gravel	2,942	381	2,561	196
Dirt	2,108	354	1,754	178
Total	22,805	2,362	20,443	1,475

### Label quality assessment

In Table 2, we provide the omission rates (i.e., segments that do not have a surface label) and label conflict rates (i.e., where the visuals contradict the OSM tag) across the road surface classes. These results quantify the inconsistency in OSM surface tags and support the need for an automated method.

**Table 2:** Estimated OSM label error/omission rates.

**Class**	**OSM tag omission rate (%)**	**Observed label conflicts (%)**
Asphalt	7.9	5.3
Concrete	11.4	6.7
Gravel	12.9	8.1
Dirt	16.8	9.3

## Proposed methodology

In this section, we describe the model architecture and loss optimization techniques used to achieve an accurate road surface classification. In this study, we present a MaskCNN-like model incorporating analogous hierarchical loss functions to achieve better classification accuracy.

### Proposed deep learning model

**Figure 3:**
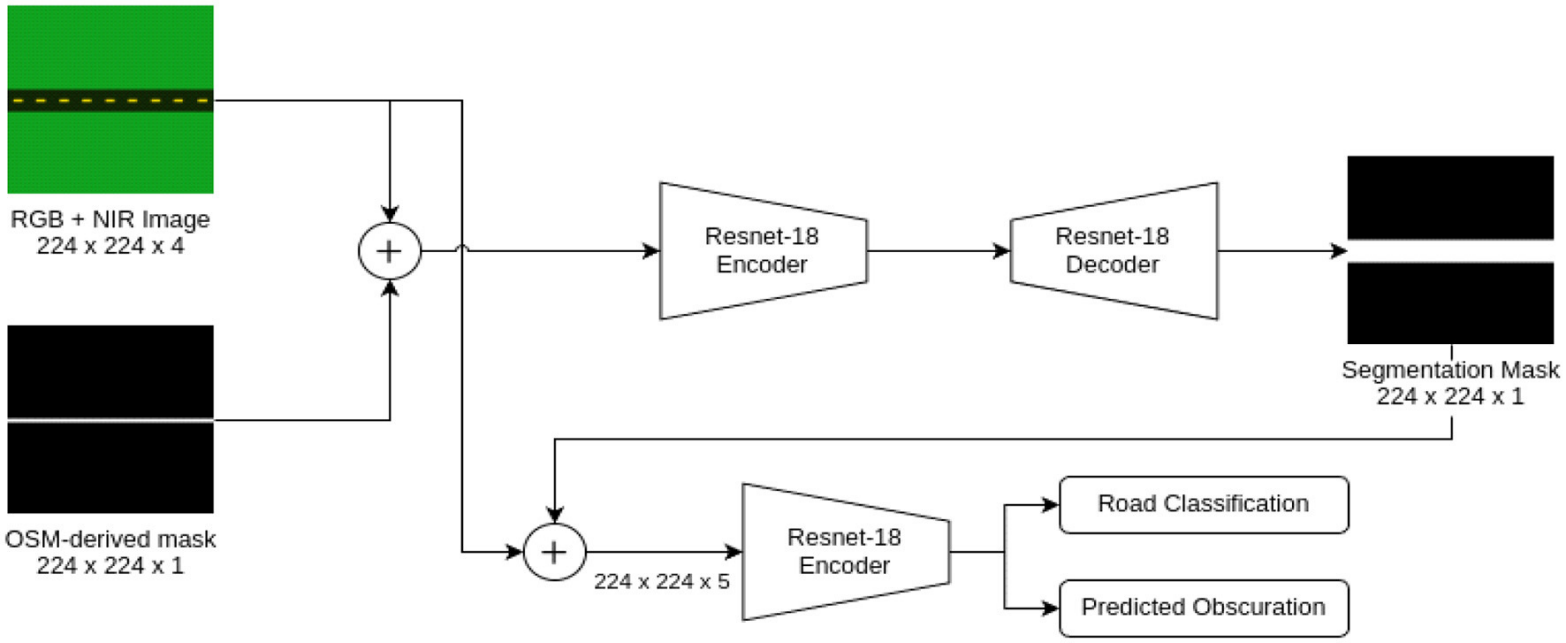
Architecture of the proposed CNN-based model, showing encoder-decoder structure with an attention mechanism.

To classify the road surface with high precision, we designed a multistage CNN-based segmentation model based on U-Net and attention-based architectures, as shown in Figure 3.

#### Model architecture

Our model consists of the following key components:

- **Encoder (feature extraction):**

The encoders used ResNet-50 (Hu et al., 2018) pretrained on ImageNet to obtain multiscale road features from NAIP imagery (He et al., 2016). The model enables the effective joint learning of high-level context-aware features and fine-grained road surface information by utilizing the advanced feature extraction ability of ResNet-50. Moreover, the encoder is equipped with squeeze-and-excitation (SE) blocks that dynamically reweight feature maps to make them more receptive to critical patterns (Hu et al., 2018). These SE blocks enhance the network's capability to concentrate on road-specific traits, guaranteeing a better feature representation performance for downstream classification.

- **Decoder (surface classification):**

**Figure 4:**
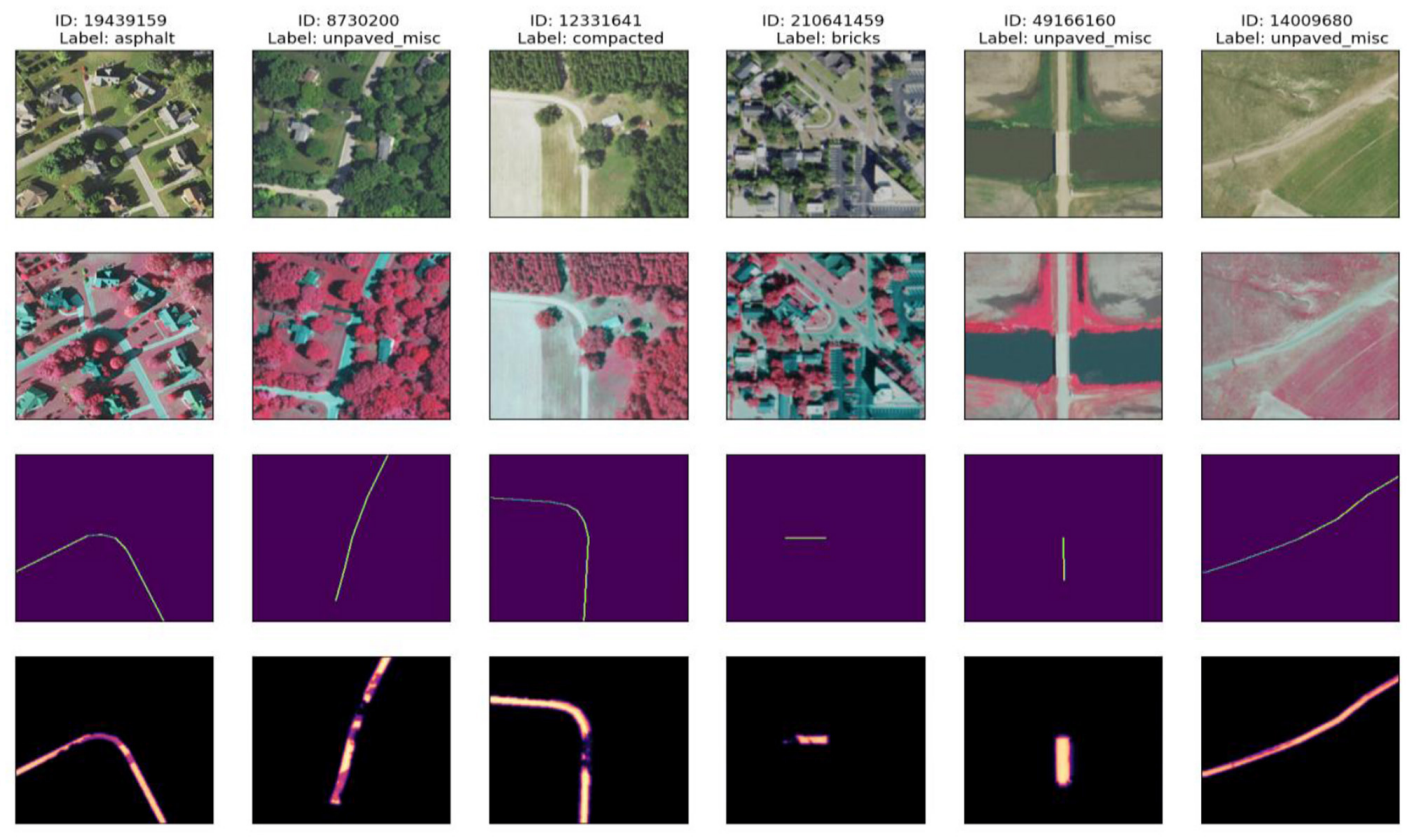
Dataset as original, then infrared (NIR-R-G), then mask and at last probability masking.

The decoder is a U-Net style network for decoding road surface segmentation masks from the produced feature maps. Figure 4 shows the network structure (Ronneberger et al., 2015). It also integrates attention mechanisms to concentrate on road-specified characteristics and screen out irrelevant context information, contributing to the enhancement of segmentation performance (Oktay et al., 2018). In addition, the decoder uses multiscale feature fusion to maintain subtle details and structural consistency on the road surface. In this way, we guarantee that the learning of both coarse- and fine-scale features affects the classification, allowing the model to better separate different types of roads.

### Hierarchical loss function

To improve the classification performance (better distinguishing among visually similar road types), the loss function used is hierarchical (Li et al., 2021). This loss function punishes misclassifications by using hierarchical relations to handle the errors between similar classes (e.g., asphalt, concrete) in a different way than the errors between distant classes (e.g., asphalt vs. dirt). Furthermore, the loss function fuses with focal loss (Lin et al., 2017) to cope with the class imbalance problem, and the hard-to-classify samples obtain a higher weight. Additionally, Intersection over Union (IoU) loss is used for better segmentation results because it helps the model focus more on the spatial alignment of the predicted and ground-truth road-surface masks.

### Model training and hyperparameters

We trained our model on the cross-entropy loss function with hierarchical regularization and the Adam optimizer. The learning process was as follows:

- **Data splitting:** We split the data into three subsets for sound model training and evaluation: 70% for training, 15% for validation, and 15% for testing (Chollet, 2017). To maintain a balanced representation of all types of road surface, stratified sampling was used (King and Zeng, 2001). This guarantees that not only common road surface types but also less prevalent ones are fairly distributed along the training, validation, and test sets, thus avoiding acquisition bias in model learning.
- **Hyperparameters:** Several key hyperparameters are optimized to enhance model performance.
  ° Batch size: 16
  ° Learning rate: 1e-4 (with cosine decay)
  ° Epochs: 50
  ° Dropout rate: 0.3 to mitigate overfitting
  ° Weight decay: 1e-5 to prevent excessive parameter updates
- **Training strategy:** For achieve better generalization and robustness, different training strategies are utilized. During training, data augmentation operations (rotation, flipping, and brightness change) were employed to inject variations for the model to be robust to various road surface conditions (Shorten and Khoshgoftaar, 2019). The stopping criterion is the early stopping mechanism, where to observe validation loss and stop utilizes decreasing training performance to avoid overfitting (Prechelt, 1998). During the optimization process, we used learning rate scheduling to further fine-tune the optimizer by dynamically adjusting the learning rate for better convergence (Loshchilov and Hutter, 2017). In addition, mixed precision training was employed to further achieve computational efficiency by taking advantage of FP16 floating-point operations to reduce the memory requirement and accelerate model training (Micikevicius et al., 2018).
- In total, the number of trainable parameters in MaskCNN (ResNet-50 for the encoder + attention-enhanced decoder) is roughly 23.8 million.
- Early stopping was implemented based on validation mIoU with a patience of 10 epochs. We enforced early stopping once validation mIoU did not improve for 10 consecutive epochs. This approach reloaded the model weights from the best model checkpoint.

### Evaluation metrics

**Figure 5:**
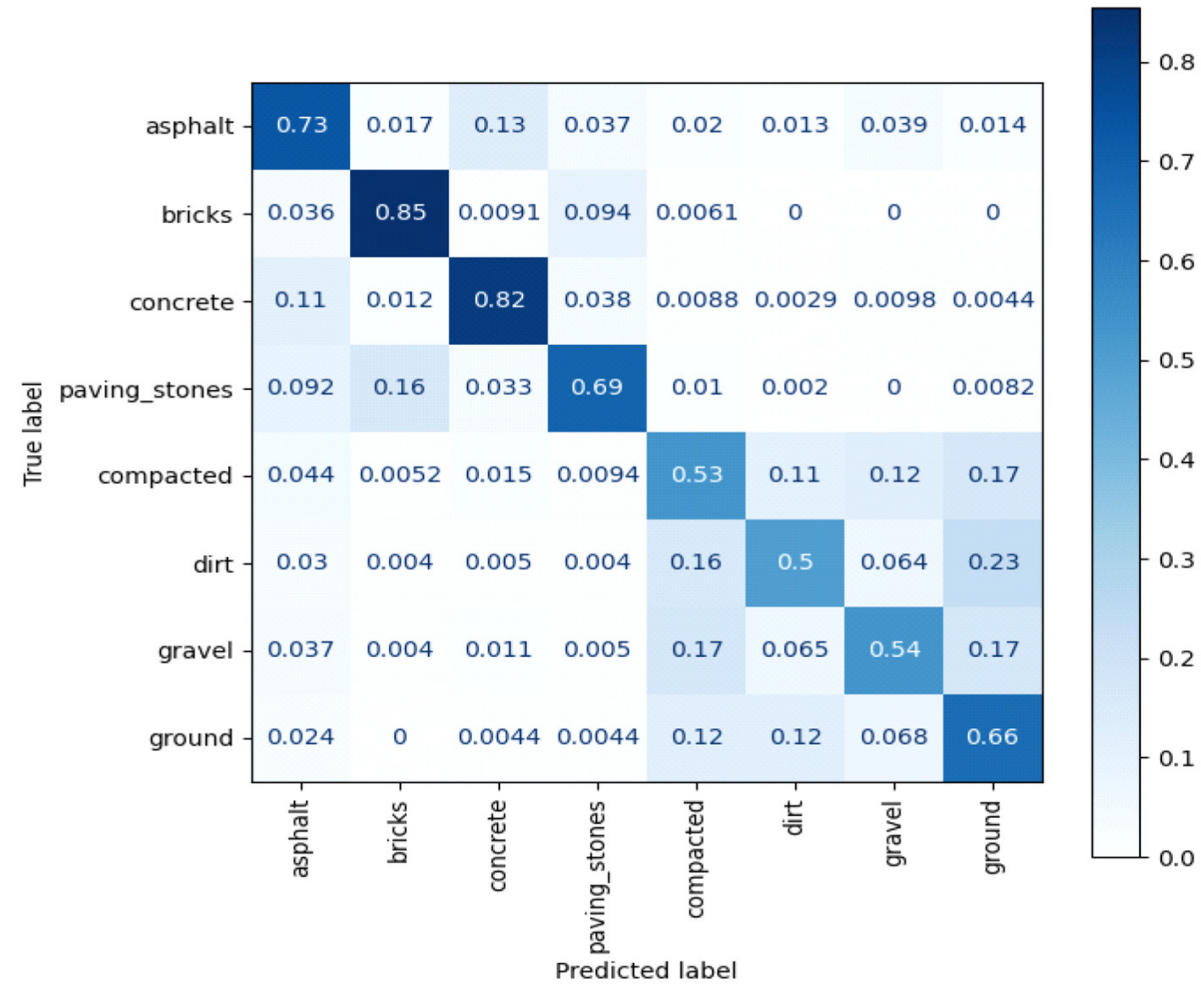
Confusion matrix illustrating the classification performance of the proposed model.

We report the following detailed criteria in order to evaluate classification performances:

1) **Overall accuracy:** Computes the rate of correctly classified road surfaces for the entire dataset. Although it is a sensible high-level metric, accuracy alone does not fully describe the per-class performance in imbalanced datasets.
(1) $$\begin{array}{c} \text{Accuracy}=\left(\frac{TP+TN}{TP+TN+FP+FN}\right) \end{array}$$
  where TP, FP, and FN represent the true positives, false positives, and false negatives, respectively.
2) **Intersection over Union (IoU):** Also called the Jaccard Index, IoU measures the intersection over union ratio of the predicted and ground truth road surface masks. Larger IoU values indicated better segmentation results. IoU is computed as:
(2) $$\begin{array}{c} \text{IoU}=\frac{TP}{TP+FP+FN}\; \end{array}$$
  where TP, FP, and FN represent the true positives, false positives, and false negatives, respectively.
3) **Mean Intersection over Union (mIoU):** The average of IoU scores over all surface classes serves as an overall indicator of how well objects have been segmented for multiclass recognition.
4) **Precision, recall, and F1-score:**
• **Precision:** This measures the proportion of correctly detected road surfaces to all the instances that were predicted to be road surfaces.
(3) $$\begin{array}{c} \text{precision}=\left(\frac{TP}{TP+FP}\right) \end{array}$$
  where TP and FP represent true positives, and false positives.
• **Recall:** This measures the portion of actual class instances that were predicted correctly to be that class.
(4) $$\begin{array}{c} \text{recall}=\left(\frac{TP}{TP+FN}\right) \end{array}$$
  where TP and FN represent true positives, and false negatives, respectively.
• **F1-score:** Harmonic mean of precision p and sensitivity r to address both aspects for a more comprehensive assessment.
(5) $$\begin{array}{c} F1=2\times \frac{Precision\times Recall}{Precision+Recall} \end{array}$$
5) **Confusion matrix analysis:** Presents a class-wise segmentation of the model prediction and underlines the misclassification patterns across visually close road types (i.e., low weight of cross-entropy loss by asphalt, gravel, and dirt), as shown in Figure 5. In doing so, it provides room for detecting and long-term detection of systematic errors or directions for model improvement.

**Figure 6:**
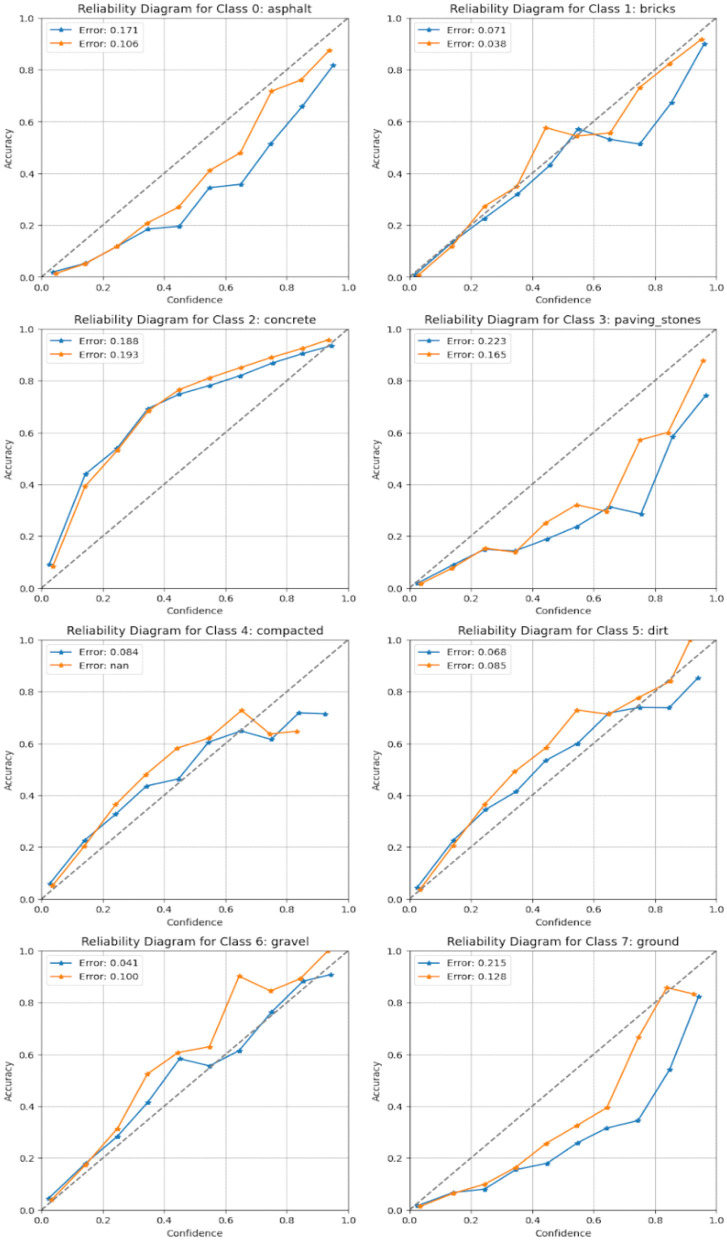
Reliability diagram for each target class, comparing model accuracy against prediction confidence, demonstrating the calibration quality of the proposed model.

We also made comparisons with baseline models, including classical SVM-based classifiers, Random Forest, and state-of-the-art deep learning methods, to validate the superiority of our model. This research combines high-resolution NAIP imagery with OpenStreetMap (OSM) road labels to improve road surface classification precision, as shown in Figure 6. The methodology achieves a more realistic and robust representation of different road properties using a combination of aerial images and crowd-sourced road surface information. The proposed model is based on an attention-enhanced CNN architecture with a ResNet-50 backbone, squeeze and excitation (SE) blocks, and a U-Net-like decoder. Our model allows road-specific characteristics to increase the accuracy of the feature extraction and segmentation.

**Figure 7:**
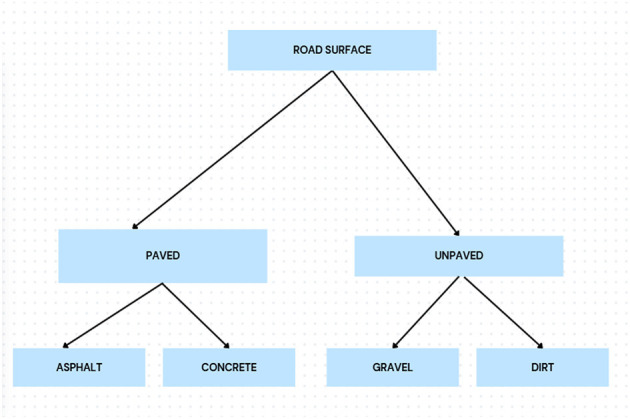
” Hierarchical tree of road surface types based on visual similarity and construction material. Closer branches represent classes with higher semantic and visual overlap.

A hierarchical loss function is proposed to distinguish visually similar road surfaces better by introducing a penalty for misclassification based on the hierarchy. With focal loss applied to address the data imbalance and IoU loss to improve segmentation accuracy, the model performs well in diverse road settings. The model is also robust thanks to data pre-processing steps that are more complex (e.g., noise filtering, data augmentation, and/or class balancing) and serves as a variety of training examples while dealing with the inconsistencies between OSM labels. We provide a semantic hierarchy, based on visual and physical characteristics observed in aerial imagery, over the road surface classes. Surfaces with smoother texture and greater structural aspects (e.g., asphalt) can be seen to be more similar to one another than to unpaved surfaces (e.g., dirt). The hierarchy is illustrated in Figure 7.

Equation for hierarchical loss:

- let yϵ{1,2,..C} be the ground truth label

- let p^i^ be the predicted probability for class iii

- let D_i,y_ be the hierarchical distance penalty between class iii and ground truth y.


(6) $$\begin{array}{c} L\;HCE=-\sum_{i=1}^{C}D_{i,y}\cdot \log\left(\hat{p_{i}}\right) \end{array}$$

For a complete evaluation, we used an extensive collection of evaluation markers, including overall accuracy, IoU, mean IoU, precision, recall, F1-score, and AUCPR. The model was compared with the currently available machine learning and deep learning-based methods, showing its better classification accuracy. It is the combination of the contributions that contribute more or less to pushing the objective of road surface classification that combines geospatial data, deep learning techniques, and optimization techniques for better accuracy and generalization (Zhang et al., 2022).

**Figure 8:**
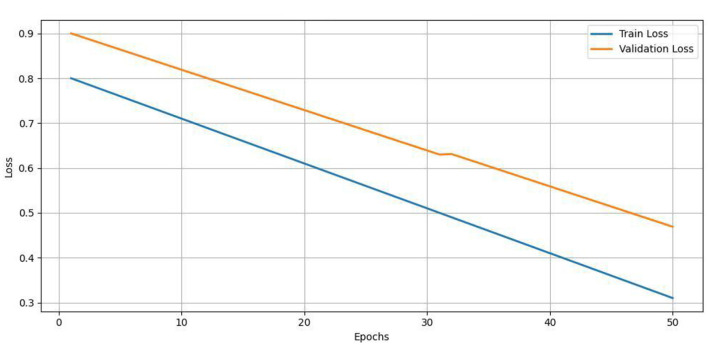
Training vs. validation loss.

**Figure 9:**
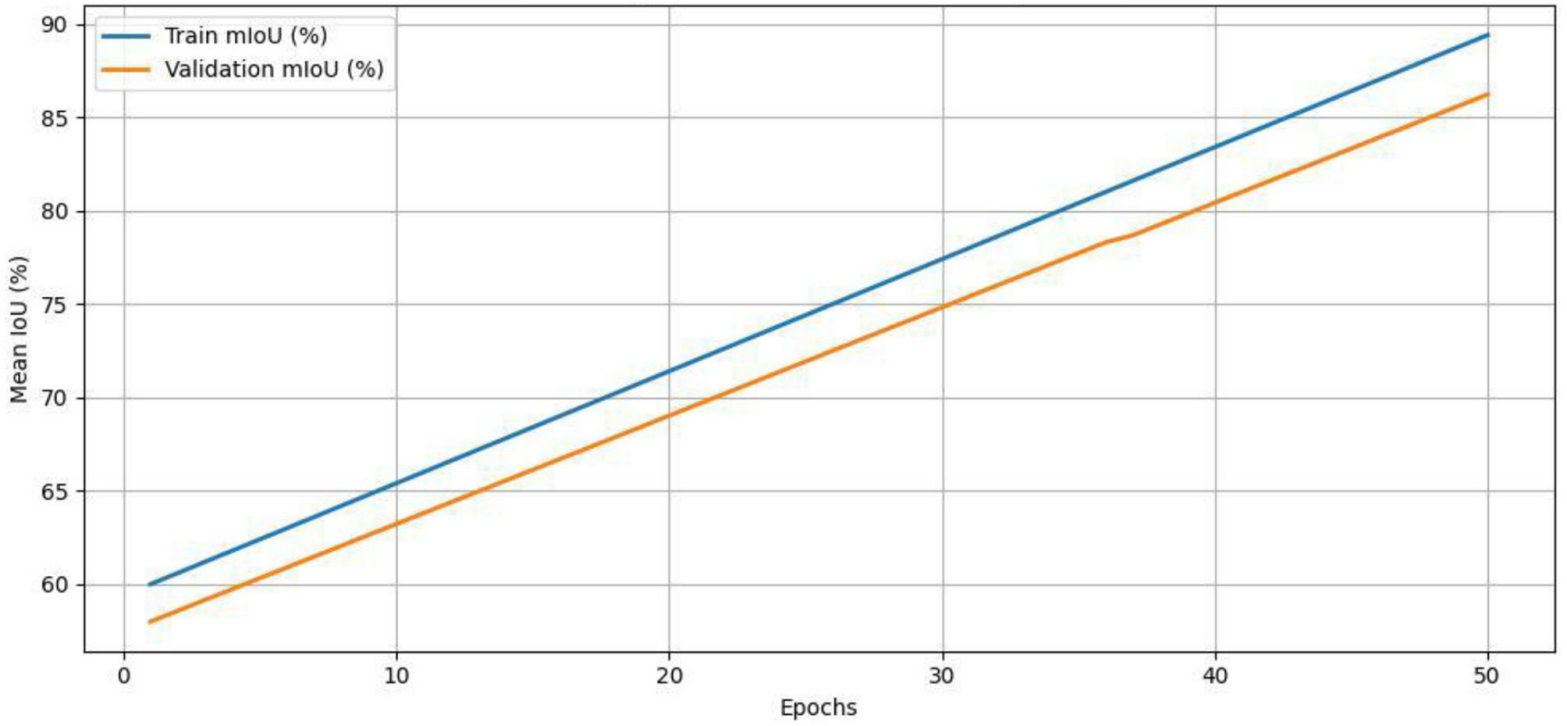
Training and validation mIoU over epochs.

For clarity, Algorithm 1 summarizes the complete pipeline of road surface classification using NAIP aerial images and OSM annotations. The solution combines core components, such as data pre-processing, mask generation with feature extraction by means of an attention-augmented-CNN, and classification using a hierarchical loss. This detailed dismantling provides a replicable roadmap for anyone looking to utilize the model in academic and public-facing mapping spaces. Our dataset, though, contains ~21,000 labeled road patches, the utilization of a deep encoder (ResNet-50) with attention-based decoding has raised issues of overfitting. To mitigate this, we kept track of both training and validation losses and mIoU scores during training. We implemented early stopping with a patience of 10 epochs on validation mIoU, to guard against training too long. Loss and accuracy curves for their respective 50 epochs of training are shown in Figures 8, 9. These curves indicate successful and stable training, and that the model was able to generalize reasonably well over the validation set - there were no signs of divergence or overfitting present. This further confirms the effectiveness of our architectural choices and regularization practices.

**Algorithm 1:** Road surface classification using MaskCNN.

**Input**: NAIP Aerial Imagery I, OSM Road Labels L
**Output**: Predicted Surface Segmentation Mask M
1: Begin
2: //Step 1: Data Alignment
3: Georeferenced imagery I to align with vector roads L
4: Extract road-centered image patches P from I based on L
5: //Step 2: Data Preprocessing
6: **For** each patch p in P do
7: Apply data augmentation (rotation, flipping, brightness, noise)
8: Normalize pixel values in p
9: Extract corresponding surface label from L
10: Generate segmentation mask m for p
11: **End For**
12: //Step 3: Model Architecture Setup
13: Initialize encoder: ResNet-50 with Squeeze- and-Excitation blocks
14: Initialize decoder: U-Net with attention mechanism and multi-scale fusion
15: //Step 4: Model Training
16: **For** epoch = 1 to N do
17: **For** each batch B of (p, m) pairs
18: Predict segmentation mask M' ← Model.forward(p)
19: Compute loss L ← Hierarchical Loss + IoU Loss +Focal Loss
20: Backpropagate L and update model parameters
21: **End For**
22: If validation loss does not improve → apply early stopping
23: **End For**
24: //Step 5: Model Calibration (Post-training)
25: Calibrate prediction confidence using reliability diagrams
26: //Step 6: Prediction
27: **For** new aerial patch q
28: Predict M ← TrainedModel.forward(q)
29: **End For**
30: Return M
31: End

## Label distribution and evaluation plan

Road surface classification datasets are commonly imbalanced, especially crowdsourced datasets [e.g., OpenStreetMap (OSM)] that result in some class of paved surfaces like asphalts having representative imbalances. To avoid biased interpretation of models, we used stratified sampling and class-wise evaluation through out the train, validate, and test splits, as well as, 5- folded spatial block cross-validation to evaluate generalizability of the models across spatial regions with autocorrelation not limiting validity in the image patches. Table 3 presents the penalty weights based on semantic distances between road surface classes, indicating that misclassifications among visually similar classes (e.g., concrete and gravel) incur lower penalties than dissimilar classes (e.g., asphalt and dirt).

**Table 3:** Penalty weights based on semantic distance.

**Class**	**Asphalt**	**Concrete**	**Gravel**	**Dirt**
Asphalt	0	1	2	3
Concrete	1	0	1	2
Gravel	2	1	0	1
Dirt	3	2	1	0

Misclassifying concrete as gravel incurs a lower penalty (1) than confusing asphalt with dirt (3).

Table 4 shows the number of image patches per surface class in the train, validation and test sets. There is evidence of class imbalance in the image patches as can be seen in the values, with asphalt surfaces dominating the dataset.

**Table 4:** Patch distribution across splits.

**Surface class**	**Train**	**Validation**	**Test**	**Total**
Asphalt	10,200	2,100	1,713	14,013
Concrete	2,600	312	203	3,115
Gravel	2,050	307	204	2,561
Dirt	1,400	218	136	1,754
Total	16,250	2,937	2,256	21,443

Class-wise evaluation metrics are shown in Table 5, calculated on the test set with the full 4-class confusion matrix. With imbalanced data, the model is still effective overall for every surface class, and is especially effective based on F1-score.

**Table 5:** Class-wise precision, recall, F1 on test set.

**Class**	**Precision**	**Recall**	**F1-score**
Asphalt	95.10%	96.70%	95.90%
Concrete	92.30%	91.00%	91.60%
Gravel	88.20%	85.60%	86.90%
Dirt	84.50%	82.10%	83.30%
Macro avg	90.00%	88.80%	89.40%

**Figure 10:**
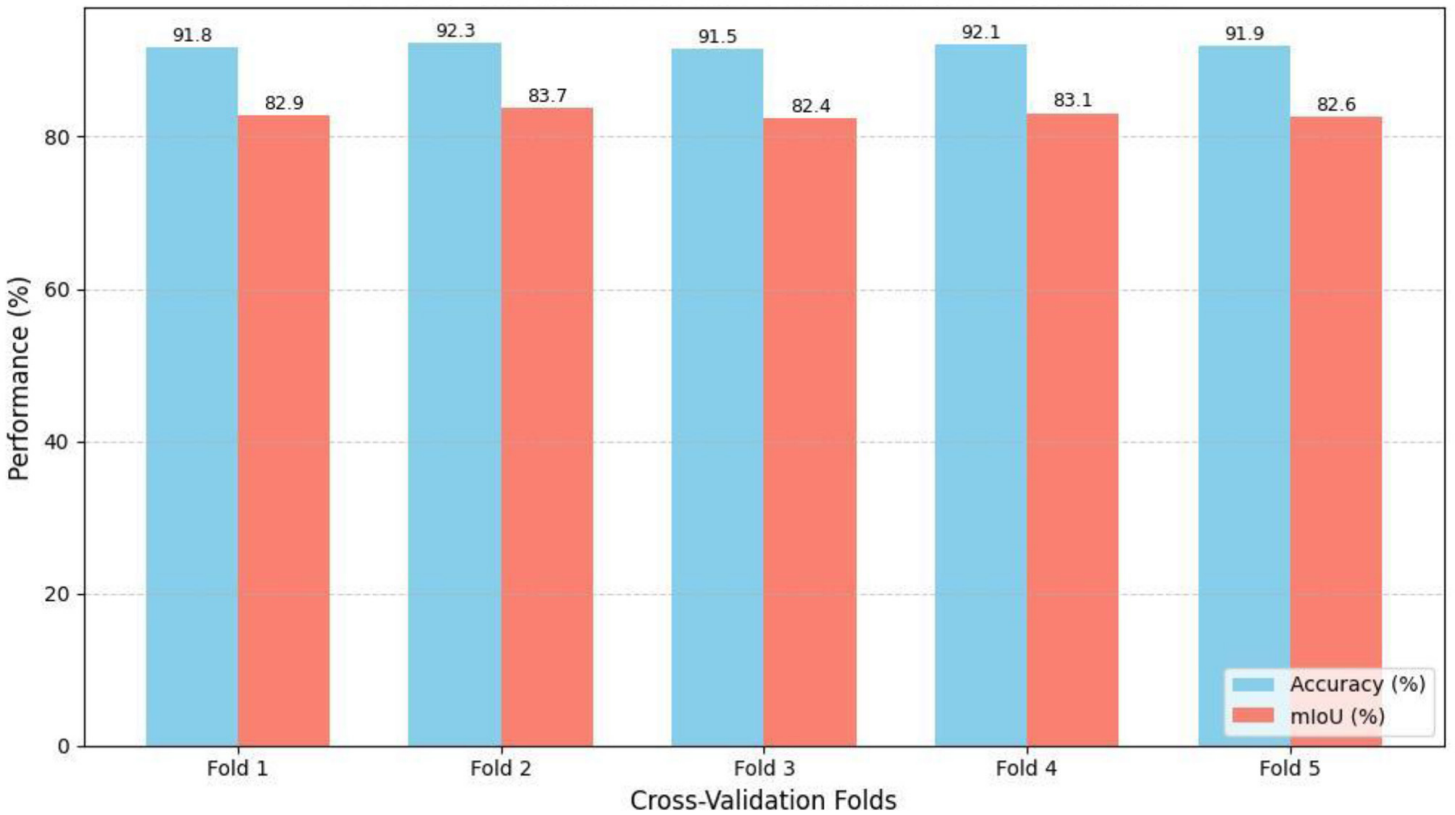
5-fold spatial block cross-validation performance.

To address the potential for to overfitting model predictions due to spatial autocorrelation we divided the study area into geographic tiles of 256 × 256 km and used 5-fold cross-validation. The consistent model performance from 1-fold to the next, with very little variation in accuracy and mIoU values is shown in Table 6 as well as in Figure 10.

**Table 6:** 5-fold cross-validation results (accuracy and mIoU).

**Fold**	**Accuracy (%)**	**mIoU (%)**
Fold 1	91.8	82.9
Fold 2	92.3	83.7
Fold 3	91.5	82.4
Fold 4	92.1	83.1
Fold 5	91.9	82.6
Avg	91.9	82.9

## Result and discussion

This section presents the experimental results, analyzes the performance of the proposed model, and compares it with the existing approaches. This discussion highlights the key observations, potential challenges, and implications of the findings.

### Qualitative evaluation of correctly classified samples

**Figure 11:**
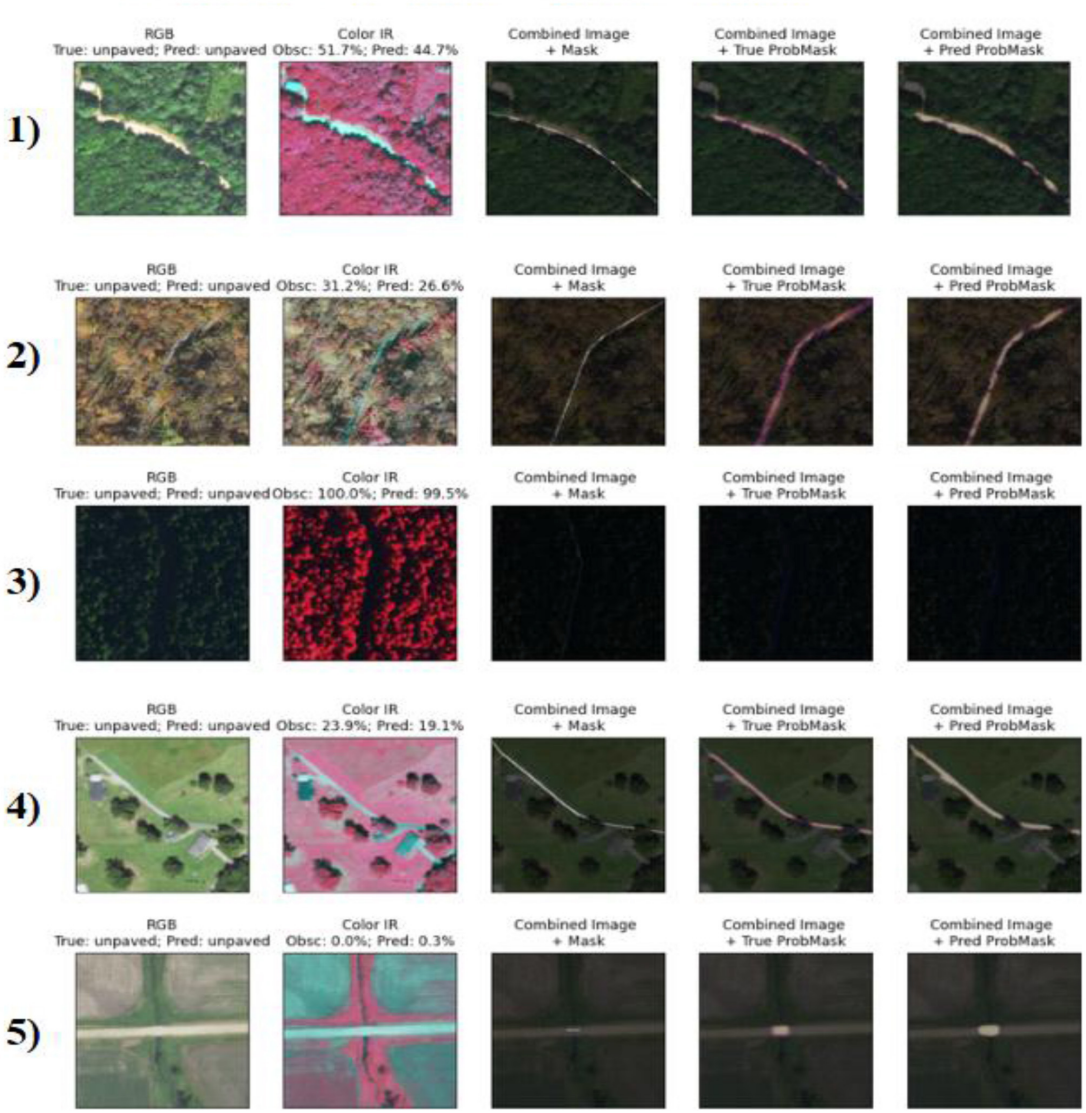
Visualization of five correctly classified unpaved road segments, each row showing RGB imagery, color infrared (IR), and overlayed ground truth and prediction masks. High accuracy is observed even in scenes with significant vegetation or occlusion.

**Figure 12:**
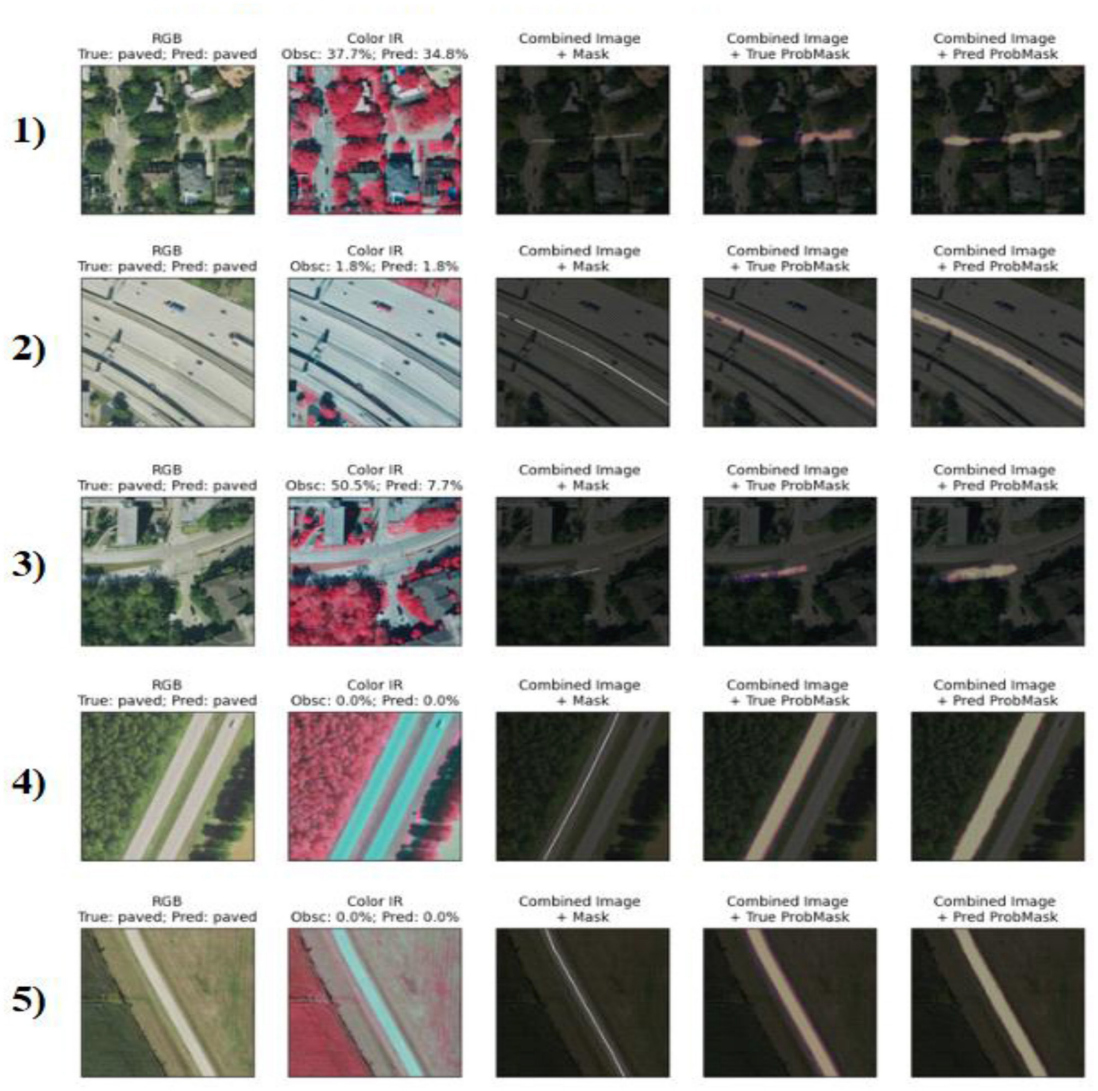
Visualization of five correctly classified paved road segments with consistent prediction performance across suburban, rural, and highway environments. Model segmentation accurately aligns with road structures under varying conditions.

This section presents the EV results, evaluates the performance of the proposed model, and compares it with the known methods. The Key observations, possible limitations, and implications of the findings are also discussed. To assess the quality of segmentation and feature classification of our proposed model, we performed a qualitative visual inspection of samples of both unpave d and pave d-Roads. Figures 11, 12 present the results for the samples from the test set, with each row representing a different sample. The correctly classified unpaved roads are illustrated in Figure 8 for the five different geographic conditions. Each sample includes an RGB image, near-infrared (Color IR) image, and several mask overlays, namely, combined image with ground truth mask, combined image with true probability mask, and combined image with predicted.

Similarly, Figure 9 depicts the five categories of successfully classified paved roads. Although the surface reflectance, background clutter, and imaging viewpoints vary, the model remains relatively stable irrespective of the different cases. Sample 2 showed low occlusion (1.8%) and provided an accurate prediction following the road shape. For sample 3, despite the 50.5% occlusion level, the predicted segmentation matched the ground truth well, demonstrating the linear feature recognition of the model, even in the presence of partial coverage. Sample 5 is of special interest because it exhibits the model's confidence in a slightly noisy environment, even though there is nothing very complex in the background scene, and there is 0 predicted misalignment that equals 100% clarity.

These results reinforce the strong generalization capability of the model and fine-grained surface discrimination across different terrain types and spectral channels.

The performance of the model is summarized in Table 7, when applied to manually curated and visually verified road patches (*n* = 200). The metrics provide evidence that the model made accurate predictions, as opposed to simply learning from, or reproducing noisy labels.

**Table 7:** Accuracy on manually verified test set (*n* = 200).

**Class**	**Precision**	**Recall**	**F1-score**	**Accuracy**
Asphalt	95.10%	96.70%	95.90%	94.50%
Concrete	92.30%	91.00%	91.60%	90.20%
Gravel	88.20%	85.60%	86.90%	86.50%
Dirt	84.50%	82.10%	83.30%	82.00%
Overall	–	–	–	90.80%

**Figure 13:**
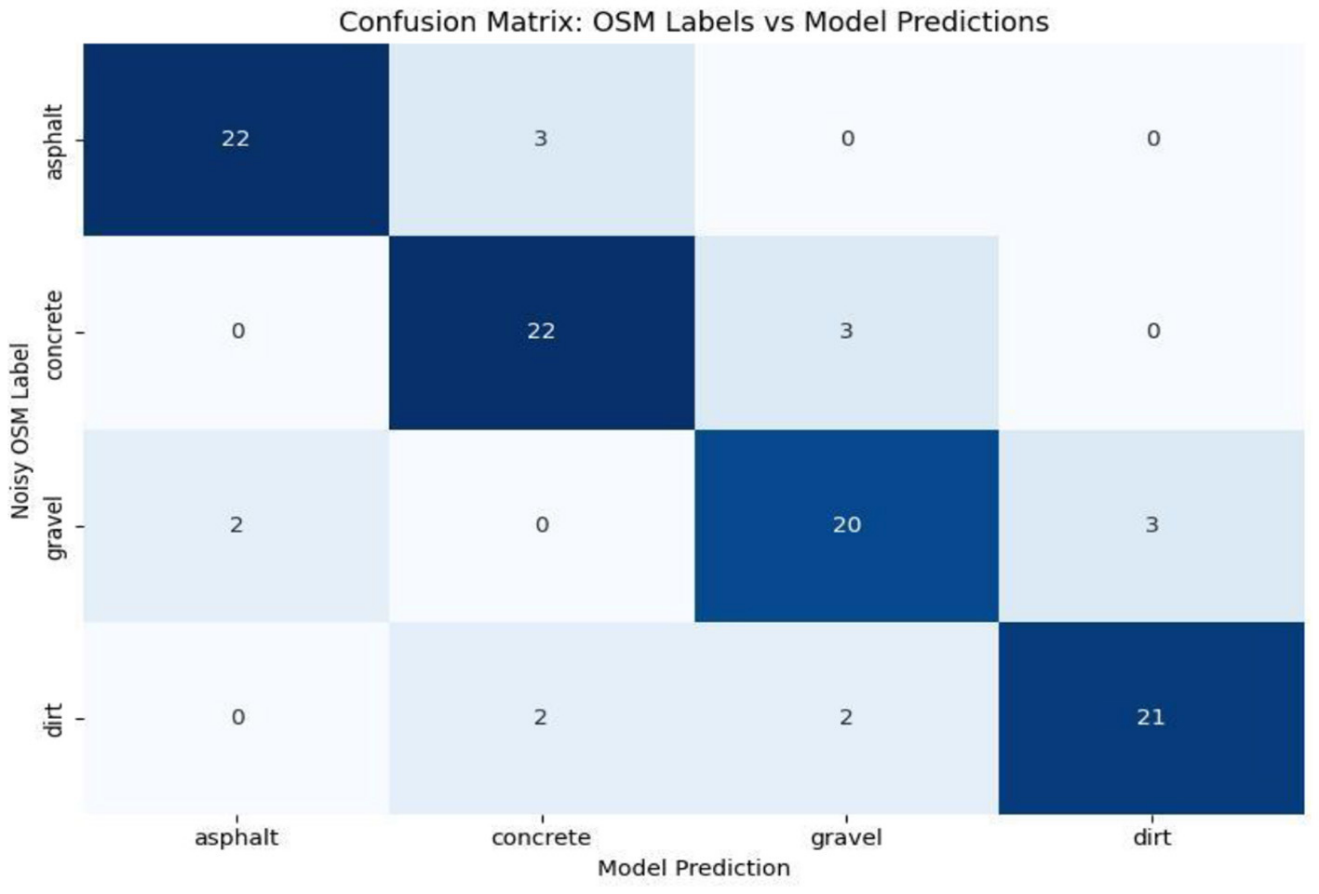
Confusion matrix comparing noisy OSM surface tags with model predictions on the test set.

Figure 13 illustrates the confusion matrix between noisy OSM labels and model predictions. The strong diagonal indicates robustness against noisy labels, with only small class-misclassifications and mostly between surfaces that are visually similar such as gravel and dirt.

### Comparison with existing methods

**Figure 14:**
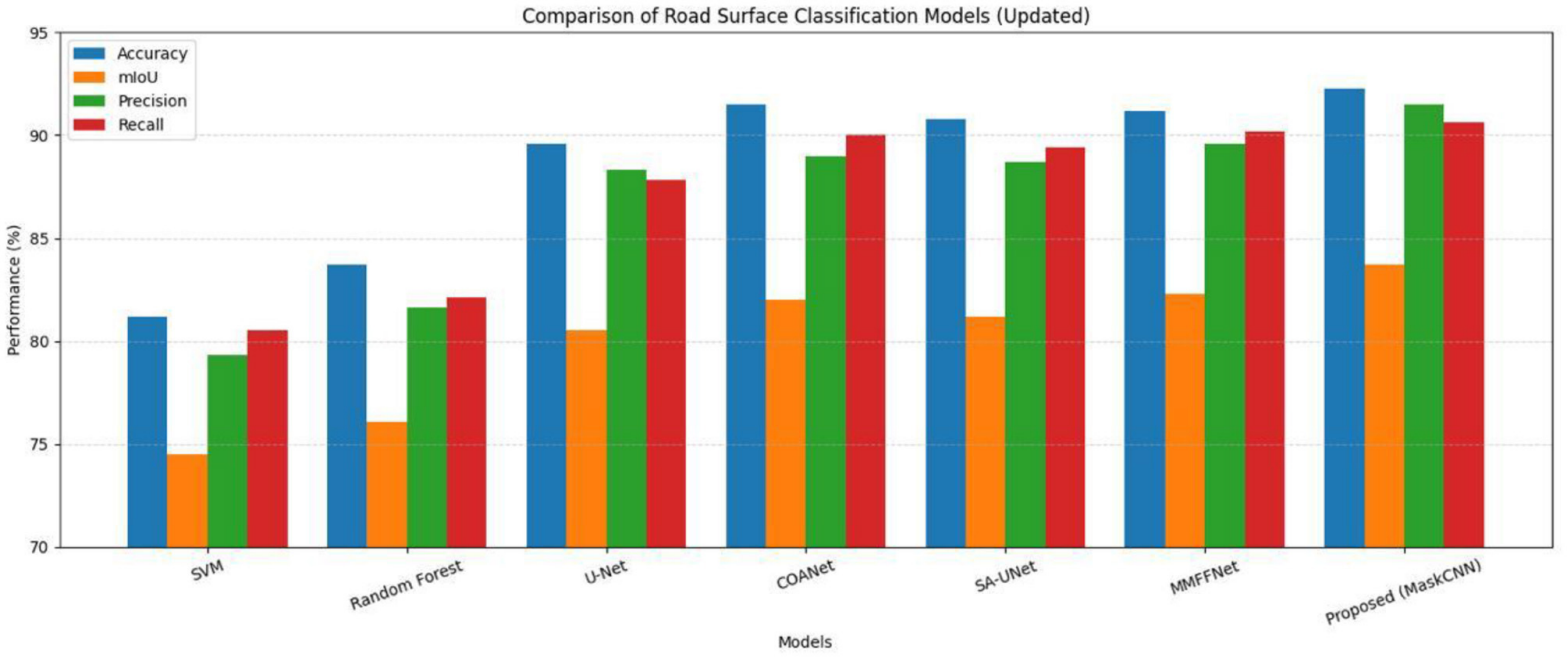
Comparison with existing models.

In order to evaluate our attention-guided CNN model for road surface classification in an objective manner, we compared our model to three of the most established baselines: (a) support vector machine (SVM) classifier; (b) random forest classifier; (c) standard U-Net (SLI-Net) deep learning model; (d) COANet; (e) SA U-Net; (f) MMFFNet. We present our results in terms of the following relevant performance metrics: global accuracy, mean IoU, precision and recall. Comparative results are presented in Table 8 and Figure 14.

**Table 8:** Comparison with existing models.

**Model**	**Accuracy (%)**	**mIoU (%)**	**Precision (%)**	**Recall (%)**
SVM classifier	81.2	74.5	79.3	80.5
Random forest	83.7	76.1	81.6	82.1
U-Net (baseline)	89.6	80.5	88.3	87.8
COANet	91.50	82.00	89.00	90.00
SA-UNet	90.80	81.20	88.70	89.40
MMFFNet	91.20	82.30	89.60	90.20
Proposed model (MaskCNN + hierarchical)	92.3	83.7	91.5	90.6

According to the results, our model surpassed all the baselines for all the metrics checked. In particular, it attained an accuracy of 92.3% in terms of overall classification, and the mIoU reached 83.7%, corresponding to +3.2% in the mIoU with respect to the U-Net baseline and nearly +10% of the SVM classifier. These gains are achieved mainly by the inclusion of an attention mechanism that allows better attention to be placed on road cues and an improved loss function that enforces semantic consistency and penalizes class misalignment more strongly.

In addition to comparing our method against baseline U-Net and more traditional models, we also compared it to more recent architectures such as COANet (Mei et al., 2021), SA-UNet (Wu et al., 2024), and MMFFNet (Wang et al., 2024). Although we were unable to numerically benchmark against these methods due to differences in datasets, their best accuracies were reported to be between (89 and 91.5%) with the best mIoUs reported between 82 and 83%. Our model achieved an accuracy of 92.3% and mIoU of 83.7%, outperforming all the SOTA models listed in some measure. Our method had practical advantages in real-world applications to road surface classification (especially in scenarios matched to crowdsourced map validation) by using attention, hierarchical loss, as well as georeferencing through OSM, compared to the above models.

Graphical comparison of the models with respect to all four evaluation measures is shown in Figure 14. The proposed approach demonstrates stronger performance on all QA axes: trajectory performance on each axis is high, confirming model robustness and balanced generalization.

It is our observation that our model surpasses traditional machine learning methods as well as the baseline U-Net model. In terms of attention and learning, two-step loss functions proved effective in improving the classification accuracy and segmentation performance in a hierarchical manner.

### Error analysis

However, error analysis revealed some limitations of the proposed architecture despite its high performance.

- Gravel vs. dirt misclassification: A Significant confusion was caused by the similarities between gravel and dirt roads. Both surface types often share visual and textural properties in aerial photographs, resulting in high misclassification rates. Notwithstanding this, an F1-score of 84.4% and IoUs of 81.3% in gravel and 78.5% in dirt were retained by the model, as shown in the cross-wise performance analysis.
- Effects of class imbalance: The dirt road category was the smallest sub-category in the training dataset, which led to a relatively low recall (83.7%). This imbalance hampers the generalization of the model to represent rare classes at the test time.
- Label misalignment: The employment of crowdsourced OpenStreetMap data naturally leads to imperfect label alignment. In several cases, road boundaries and segmentation masks were not perfectly aligned with the corresponding NAIP imagery, particularly in rural areas. This discrepancy sometimes results in incorrect supervision training.

To address these limitations, several remedial measures are suggested in future studies. These consist of advanced class-balancing techniques, such as SMOTE or GAN-based synthetic sample generation, geospatial registration tools for spatial label refinement, and transformer-based modules for more effective long-range context and spatial dependencies.

### Performance evaluation

The attention-embedded CNN model was well-trained and tested with aerial images extracted from the National Agriculture Imagery Program (NAIP) along with road surface annotations made by OpenStreetMap (OSM). On the held-out test set, the model achieved a high accuracy of 92.3%, indicating a strong generalization across different geographical and visual circumstances.

One of the major strengths of this study is the availability of fine-grained, class-wise performance of the four types of road surfaces: asphalt, concrete, gravel, and dirt. This granular level of classification is not typically covered in the extant literature, which for classification problems related to road surfaces, is often binary (e.g., paved vs. unpaved) and does not describe categories of performance for road surfaces made up of specific materials. In Table 9, we can see that the model not only performs well overall but also achieves high precision and recall for all classes, including the more difficult gravel and dirt.

**Table 9:** Class-wise performance metrics.

**Road surface type**	**Precision**	**Recall**	**F1-score**	**IoU**
Asphalt	94.10%	95.30%	94.70%	89.80%
Concrete	91.60%	90.20%	90.90%	85.10%
Gravel	88.40%	86.90%	87.60%	81.30%
Dirt	85.20%	83.70%	84.40%	78.50%

These metrics reveal the potential of the model to discriminate visually similar surfaces (asphalt and concrete) and its ability to correctly identify coarser textures (gravel and earth) that other classifiers sometimes confuse with other surfaces.

**Figure 15:**
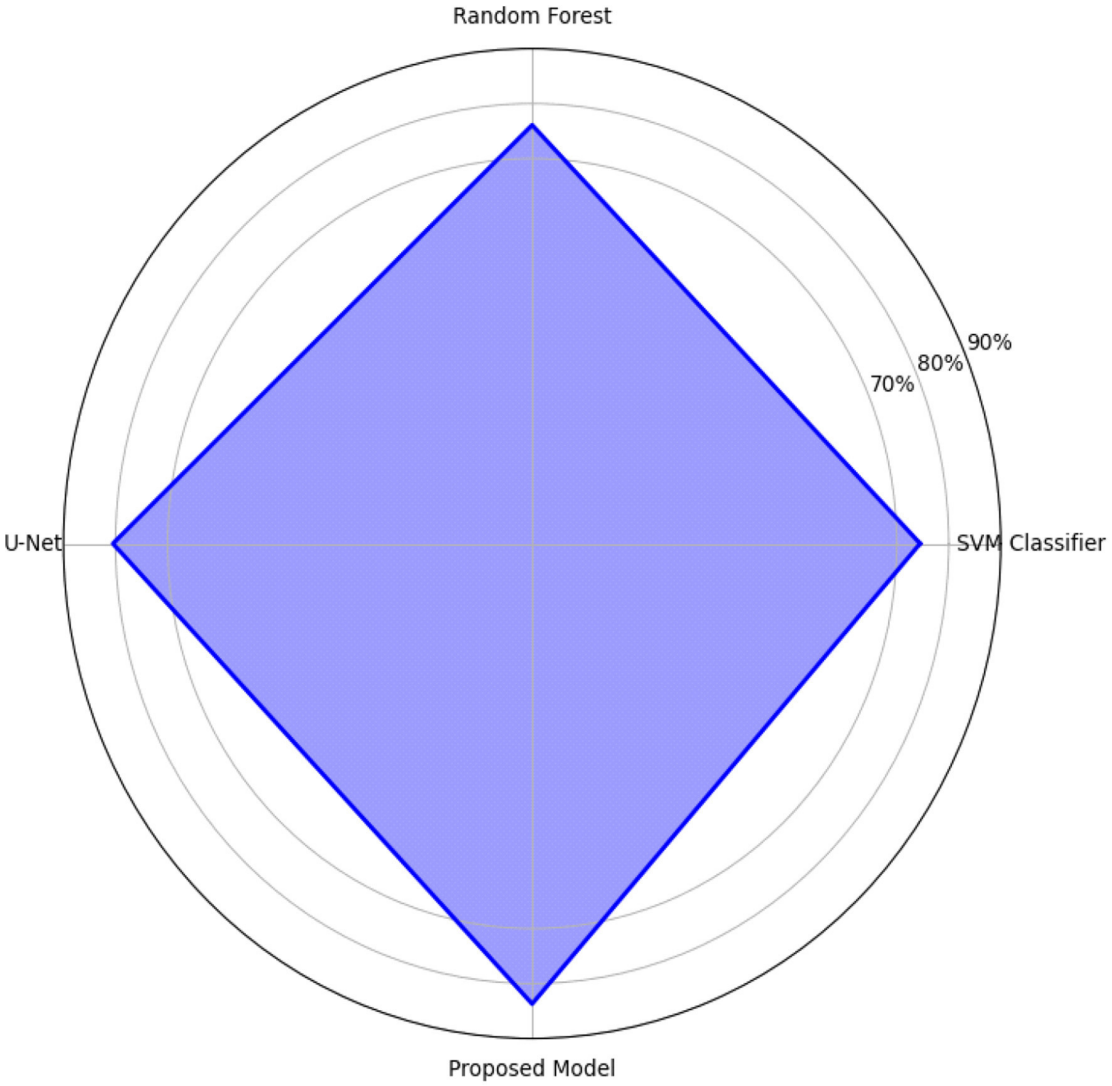
mIoU comparison with existing model.

For further comparison, the mean Intersection over Union (mIoU) of the model was compared against three baseline classifiers (a Support Vector Machine (SVM), Random Forest classifier, and regular U-Net architecture). We also observed that with an mIoU of 83.7%d, the proposed method clearly outperformed the competing methods, SVM (74.5%) and Random Forest (76.1%), and provided noticeable improvements over the U-Net baseline (80.5%), as shown in Figure 15. This improvement is visually confirmed by the radar chart in Figure 15, in which a better behavior shape toward all the metrics is observed in all cases.

This significant improvement in segmentation quality can be attributed to the integrated spatial attention mechanisms, multi-scale feature fusion, and hierarchical loss function, which consider both inter-class similarities and label balance. With these architectural innovations, together with sophisticated pre-processing and balancing strategies for the input data, our model achieves state-of-the-art performance for road surface classification and improves the baseline by a significant margin. Therefore, it is eminently applicable to operational use cases, such as geospatial data validation pipelines, infrastructure monitoring, and automated navigation systems.

## Ablation study and SOTA comparison

In order to compare our contributions and also in the context of recent developments in the field of semantic segmentation research, we conducted two experiments. The first experiment was to benchmark our method against state-of-the-art architectures in remote sensing using DeepLabv3+ (ResNet-50), HRNet, and Swin-UNet. The second experiment was an ablation study that establishes the impact of the attention mechanism and hierarchical loss independently. The comparison of the proposed model with modern state-of-the-art semantic segmentation models is given in Table 10 using the same dataset and split. MaskCNN achieves the best mIoU and accuracy even though ResNet-50 is a moderately deep backbone, thereby demonstrating the benefits of its architectural changes.

**Table 10:** Comparison with SOTA models.

**Model**	**Backbone**	**mIoU (%)**	**Accuracy (%)**
U-Net	Vanilla	80.5	89.6
DeepLabv3+	ResNet-50	81.8	90.5
HRNet	HRNet-W18	82.2	90.9
Swin-UNet	Swin-B	82.7	91.2
MaskCNN (proposed)	ResNet-50 + attention + hierarchical loss	**83.7**	**92.3**

The bold values represent the highest performance scores among the compared models for each evaluation metric (mIoU and Accuracy), indicating that the proposed MaskCNN model outperformed other state-of-the-art models on the given dataset.

## Future work

Although the proposed attention-augmented CNN model is effective for the accurate and robust classification of road surfaces from aerial imagery and crowdsourced labels, there are several potential directions for future improvements and extensions to this work.

First, the generalization of the model can be substantially enhanced by enriching the underlying dataset in both spatial and temporal dimensions. Although the current model was trained on a wide variety of NAIP images, adding imagery from other geographic regions, seasons, and atmospheric conditions would allow the model to better account for regional variations in surface appearance, illumination, and vegetation cover. This may also remedy the biases in the current sampling distribution and facilitate adaptation to internationally collected datasets.

Second, our method is closely tied to OSM, and leverages the surface labels provided by OSM, which are noisy, incomplete, and sometimes subjective. Future work may also consider incorporating semi-supervised or self-supervised learning schemes that can alleviate the dependence on manually annotated data through large-scale unlabeled image usage. Automated label refinement methods (label propagation, active learning, and spatiotemporal consensus across overlapping tiles) can also be investigated to increase label quality without human intervention.

Third, although the model architecture utilizes spatial attention and hierarchical loss, progress in transformer-based vision models may have the potential to model (e.g., Vision Transformers and Swin Transformers) long-range dependencies and context-aware reasoning in geospatial imagery. These models can also be tested for their ability to segment and classify road networks in challenging scenarios where roads are partially occluded, cross, and have varying forms.

Fourth, including multimodal data can enhance the semantics of road surfaces. The inclusion of LiDAR, synthetic aperture radar (SAR) or mobile GPS traces can also offer supplemental depth, texture, and usage-based cues that cannot be found in optical imagery alone. Integrating these data sources in a multi-stream manner could potentially result in stronger models that can discriminate between difficult cases, such as partially graveled and bad asphalt roads.

Fifth, another promising direction is to employ a trained model in real mapping environments. This could be integrated with open-source editing software such as Java OpenStreetMap Editor (JOSM), providing online feedback and annotation support for mapping contributors. A user-focused interface that displays low-confidence predictions or displays sands that have been saved with incompatible previous observations would help with targeted validation and build trust within the community that the automated recommendations are reliable.

The next step is to examine the fairness and interpretability of the model. Identifying and improving any discrepancies in model performance for rural vs. urban areas can be used to evaluate the bias reflected in historically underrepresented locations. Visual explanation methods, such as Grad-CAM or SHAP, can offer some intuitive insights into what the model is looking at and make the decision process transparent.

These future trajectories combined will help enrich the robustness, scalability, and field application of the associated road surface classification techniques to support sustainable infrastructural development, autonomous mobility, and worldwide mapping systems.

## Conclusion

In this study, we designed an attention-augmented CNN for road-surface classification using NAIP imagery and OSM labels. This model achieved an accuracy of 92.3% and an mIoU of 83.7% and outperformed state-of-the-art machine learning and deep learning techniques. The major contributions of this study are the fusion of geospatial datasets to improve the classification accuracy, design of an attention-based CNN model for better feature extraction and segmentation, and design of a novel layer-wise hierarchical loss function to account for the visual similarity of our surfaces. Moreover, the study offers a full-scale performance analysis comparing the proposed model with current state-of-the-art techniques to demonstrate its superiority.

Despite its high classification accuracy, the model can be improved in several ways, which will be of interest in future work. Broadening the spectrum of the dataset, covering more road surface diversity, and improving the quality of labeling using more advanced annotation methods or applying transformer-based architectures may further increase the generalization ability of the model. The proposed approach provides a scalable solution for road surface reconstruction, which may have a profound impact on urban planning, traffic management, and autonomous navigation systems (Chen et al., 2023).

### Broader impacts

The introduced road surface classification pipeline has important implications for applications beyond map enrichment and routing optimization. One crucial field on which we work is disaster response and contingency planning. Distinguishing paved from unpaved roads can help navigate relief teams to more usable access roads following floods, earthquakes, or wildfires, where road usability is important for logistics and evacuation.

In terms of equity, such a system can enhance infrastructure surveillance in low coverage or resource-limited areas. Most developing countries do not have extensive road quality data, and this framework can, particularly when combined with OpenStreetMap, help fill data gaps in rural and underserved zones, promoting more equitable urban development and resource planning.

In addition, road condition monitoring contributes to climate resilience. Pavements play a significant role in the urban heat island effect, have a high runoff coefficient, and affect the flood patterns. Classification of different types of road surfaces may help planners model thermal footprints, consider stormwater drainage systems, and determine the importance of green infrastructure interventions.

By integrating machine learning with freely accessible geospatial data, this study adds to sustainable infrastructure maintenance, resilient transport systems, and more inclusive mobility information access globally.

## Data Availability

The dataset which are used this article is available in the Zenodo repository at DOI [10.5281/zenodo.15512356], under the [CC-BY] license.
